# Supplementary figures and images for: HOPS/CORVET tethering complexes are critical for endocytosis and protein trafficking to invasion related organelles in malaria parasites
Source: PLoS Pathog. 2025 Apr 8;21(4):e1013053. doi: 10.1371/journal.ppat.1013053 (PMC12011295; doi:10.1371/journal.ppat.1013053)

S1 Fig

**A**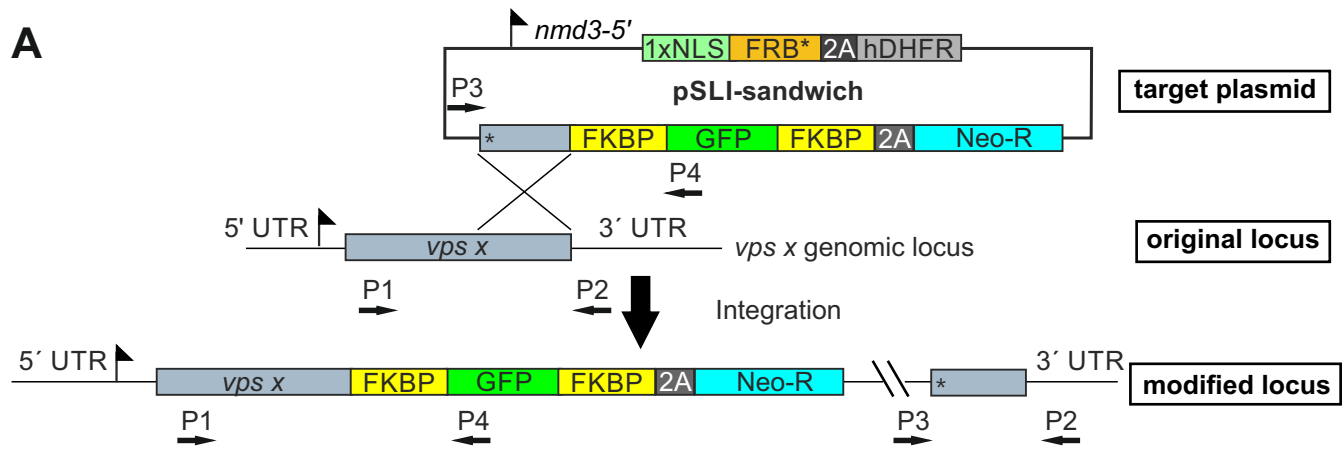**B**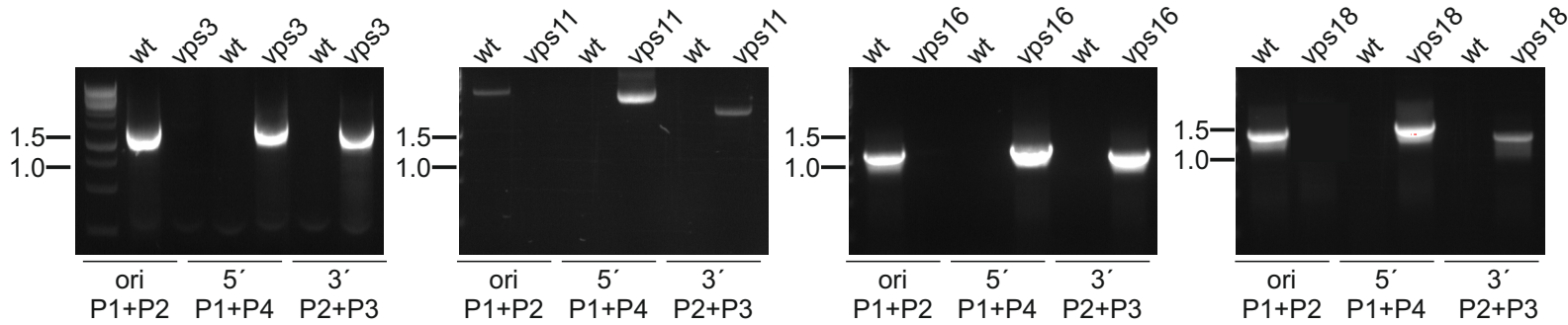**D**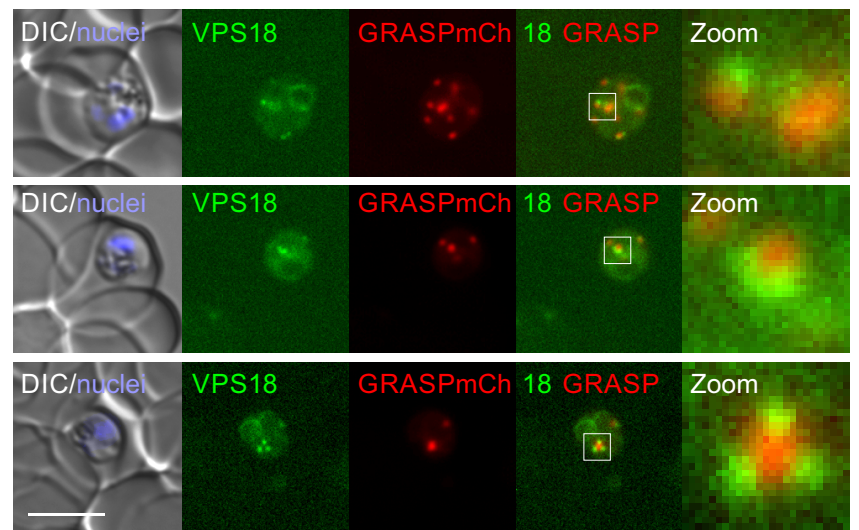**C**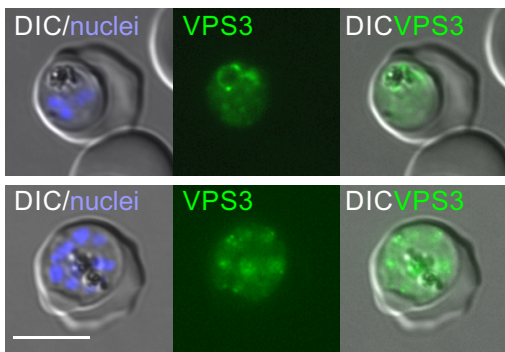**E**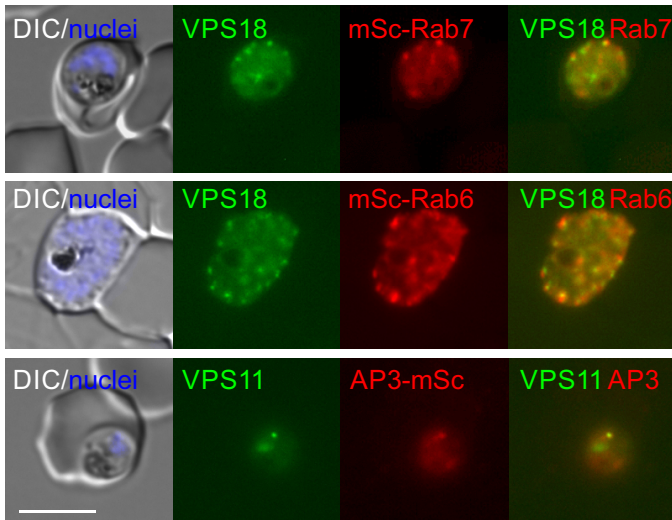**F**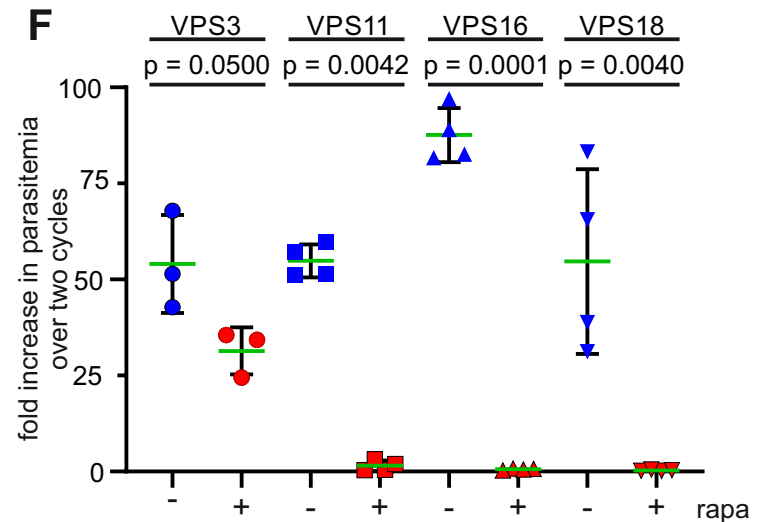

Supplement: S1 Fig — (A) Detailed schematic representation of the SLI strategy to modify the genomic locus of the core subunits of HOPS/CORVET and to express simultaneously a nuclear mislocalizer for knock sideways. Binding sites of the oligonucleotides chosen for validation of genomic integration are shown (P1-4). FKBP: FK506 binding protein, dimerization domain; 2A: skip peptide; Neo-R: neomycin phosphotransferase; hDHFR: human dihydrofolate reductase; NLS: nuclear localization signal; FRB: FKBP-rapamycin-binding domain. (B) Confirmatory PCR on genomic DNA from 3D7 (wt) and the indicated cell lines to validate correct integration of the SLI plasmid at the 3’- and 5’-end and disruption of the original locus (ori) using combinations of the primers (P1-4) indicated in A. Size in kbp is shown. (C) Live-cell fluorescence microscopy images of transgenic parasites expressing the endogenously 2xFKBP-GFP-2xFKBP tagged VPS3 in trophozoites (upper panel) and late stages (lower panel). Nuclei were stained with DAPI. DIC, differential interference contrast. Scale bars: 5 μm. (D) Live cell fluorescence microscopy images of transgenic parasite lines expressing endogenously tagged VPS18-2xFKBP-GFP together with GRASP-mCherry as marker for the Golgi apparatus. Nuclei were stained with DAPI. DIC, differential interference contrast. Scale bars: 5 μm. Zoom (300x) of the indicated white boxes is shown to visualise localization of the VPS18 vesicle-like foci in relation to Golgi. (E) Live cell fluorescence images of VPS18-2xFKBP-GFP-2xFKBP parasites co-expressing mScarlet- Rab7 (late endosomes, ELC) and mScarlet-Rab6 (trans-Golgi) and VPS11-2xFKBP-GFP-2xFKBP parasites co-expressing AP3-mScarlet. Scale bars: 5 μm (F) Growth rate (fold increase in parasitemia versus start parasitemia) over two replication cycles of the indicated cell lines in presence (+, red) or absence (−, blue) of rapalog (rapa) calculated from the curves in Fig 1D. Mean (green line) of n = 4 for VPS11, 16 and 18 and n = 3 for VPS3. Error [file ppat.1013053.s001.pdf]

S2 Fig

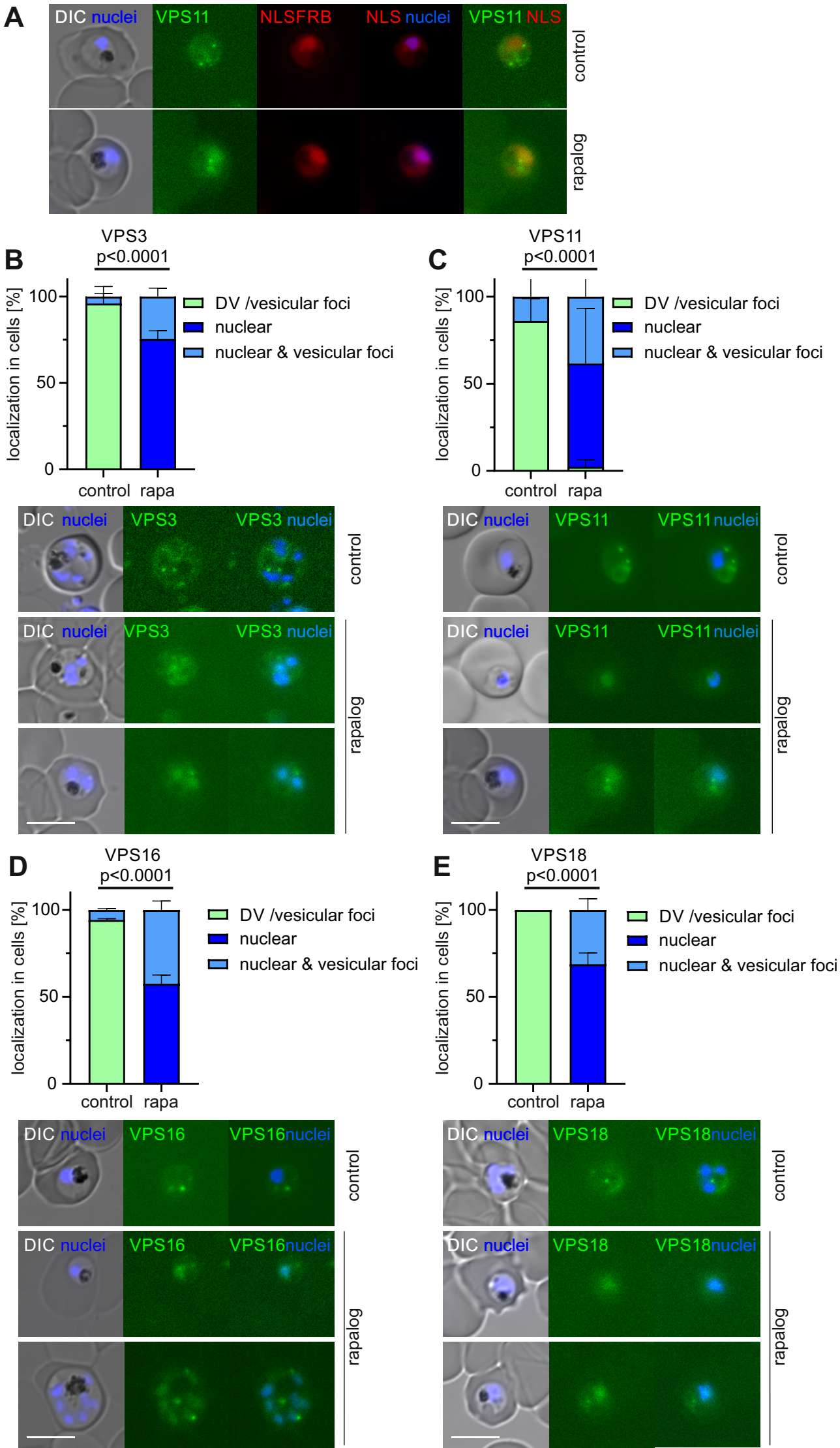

Supplement: S2 Fig — (A) Live fluorescence images of control and KS-induced (rapalog) VPS11-2xFKBP-GFP-2xFKBP parasites co-expressing 1xNLS-FRB-mCherry (B-E) Upper panel, quantification of the number of cells showing a digestive vacuole (DV)/ vesicular foci (wild type), nuclear (mislocalized) and nuclear/vesicular foci (partially mislocalized) localization of the indicated VPS subunits in control and KS-induced (rapa) parasites. Results from n = 2 independent replicates with a total of 136 (control) and 123 (rapalog) VPS11 parasites; 136 (control) and 126 (rapalog) VPS16 parasites; 88 (control) and 88 (rapalog) VPS18 parasites. p values from a Chi-square test are indicated. Lower panel, representative fluorescence live cell images of control and KS-induced (rapalog) parasites. (PDF) [file ppat.1013053.s002.pdf]

S3 Fig

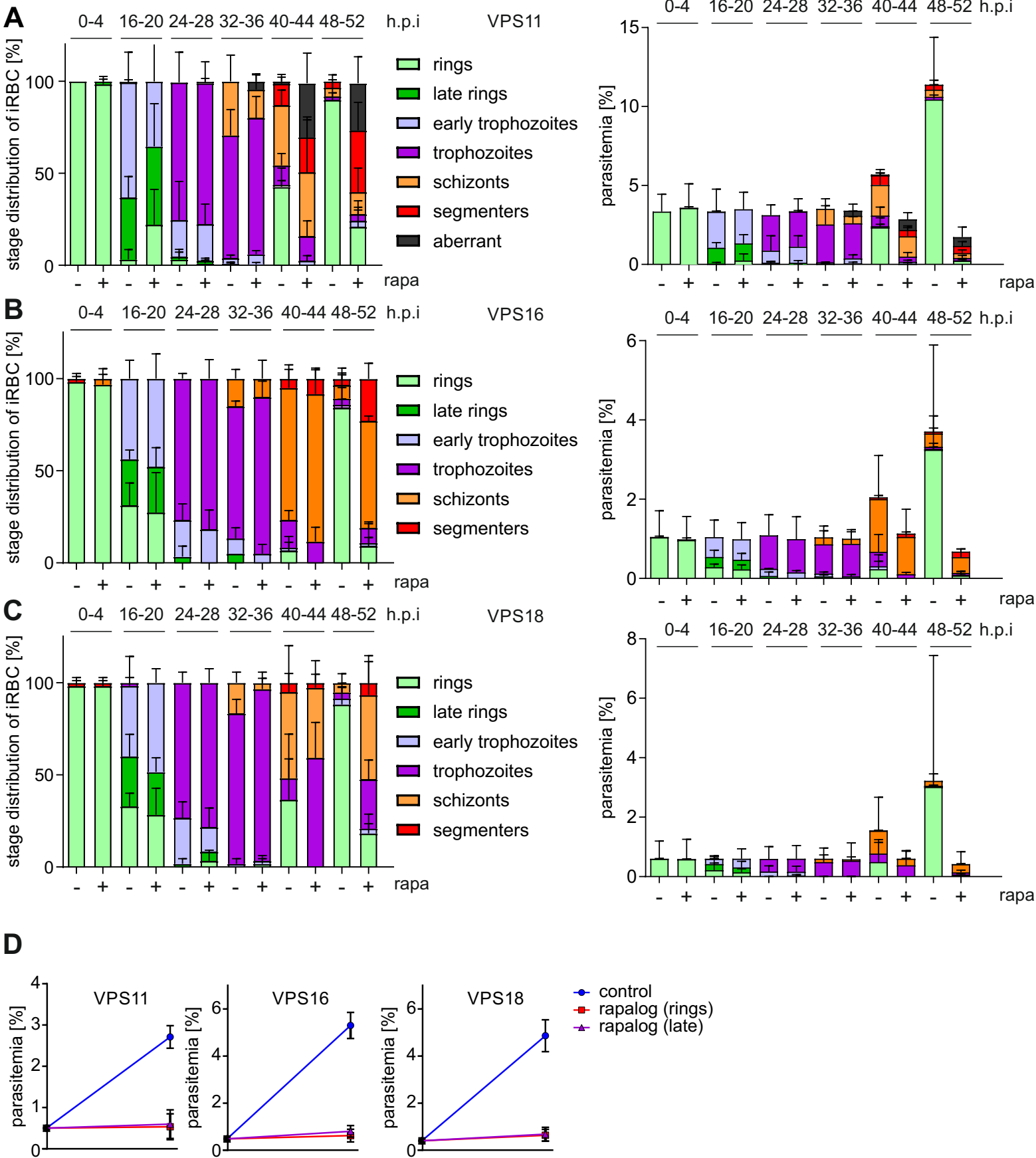

Supplement: S3 Fig — (A, B, C) Stage distribution of control (−) and KS-induced parasites (+ rapa). Left, stage distribution relative to the total of counted infected red blood cells at the indicated time points (h.p.i). Right, stage distribution relative to the total parasitemia at the indicated time points. Mean of n = 3 independent replicates with at least 50 parasites per time point. Error bars indicate SD. (D) Flow cytometry-based determination of parasitemia of synchronised cultures of the indicated parasite lines after one replication cycle without rapalog (control) or when rapalog was added to the rings (0–4 h.p.i, red line) or to late stages (32–36 h.p.i, purple). (PDF) [file ppat.1013053.s003.pdf]

S4 Fig

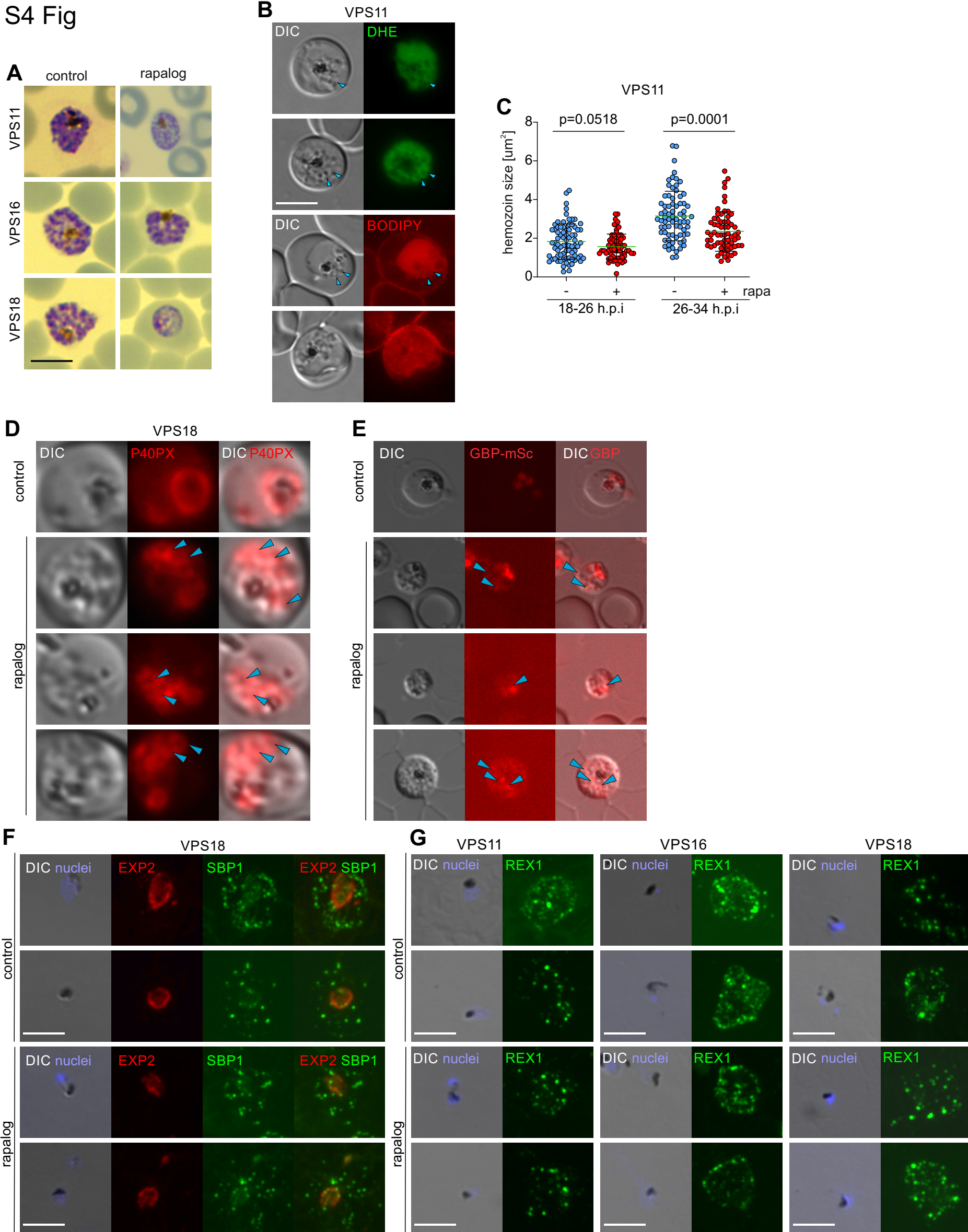

Supplement: S4 Fig — (A) Images of Giemsa smears of VPS11, 16 and 18 control and KS-induced parasites (rapalog) when rapalog was added to the ring stages. Scale bars: 3 μm. (B) Images of DHE (upper panel) and BODIPY-stained (lower panel) control and KS-induced (rapalog) VPS11 parasites. Blue arrows show vesicles. Scale bars: 5 μm. (C) Hemozoin size (µm2) of control (- rapa) and KS-induced (+rapa) VPS11-2xFKBP-GFP-2xFKBP parasites at the indicated time points. Mean (green line) of n = 3 independent replicates with a total of 77 control and 66 KS-induced parasites at 18–26 h.p.i and 73 control and 65 KS-induced parasites at 26–34 h.p.i. Error bars indicate SD and p values from a two-tailed unpaired t-test are indicated. (D) Enlarged micrographs of control and KS-induced (rapalog) VPS18-2xFKBP-GFP-2xFKBP parasites expressing P40PX-mCherry. Blue arrows show vesicles surrounded by P40PX. Zoom factor 65x (E) Live cell images of saponin-lysed control and KS-induced (rapalog) VPS18-2xFKBP-GFP-2xFKBP parasites expressing GBP1-108-mScarlet. Blue arrows show overlapping of vesicles and mScarlet signal. Zoom factor 600 x (F) IFA images of control and KS-induced (rapalog) VPS18-trophozoites probed with α-EXP2 (parasitophorous vacuole membrane resident protein) and α-SBP1 (Maurer´s clefts resident exported protein). Nuclei stained with DAPI. DIC, differential interference contrast. Scale bars: 5 μm. (G) IFA images of control and KS-induced (rapalog) trophozoites of the indicated cell lines probed with α-REX1 (Maurer´s clefts resident exported protein). (PDF) [file ppat.1013053.s004.pdf]

S5 Fig

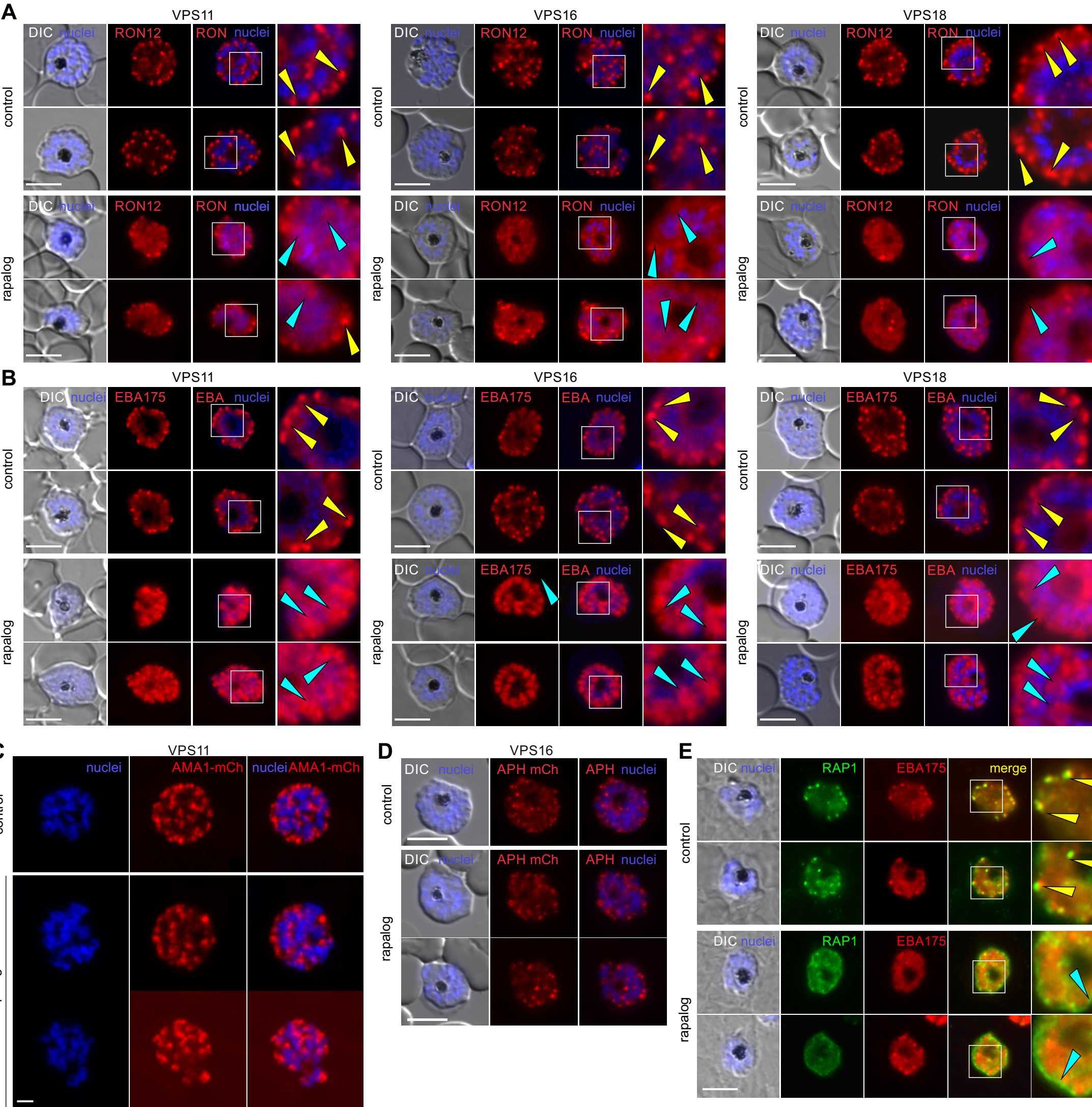

Supplement: S5 Fig — (A, B) Representative live-cell fluorescence microscopy images of C2-arrested control and KS-induced (rapalog) VPS11 (left) VPS 16 (middle panel) and VPS18 (right) schizonts expressing RON12-mCherry (rhoptry neck luminal protein) (A) and EBA175-mCherry (transmembrane microneme protein) (B). Scale bars: 5 μm. Enlarged micrographs (zoom: 600x) of the indicated white boxes are shown right of each panel to visualise localization of organelle markers. Yellow arrows show a typical microneme or rhoptry apical localization, light blue arrows show an aberrant localization: diffuse around and in the merozoites, PV (RON12) or around the nucleus. Nuclei stained with DAPI. DIC, differential interference contrast. Scale bars: 5 μm. (C) Confocal live-cell fluorescence microscopy images of C2-arrested control and KS-induced (rapalog) schizonts of VPS11 parasites expressing AMA1-mCherry. Nuclei stained with DAPI. Scale bars: 2 μm. (D) Live-cell fluorescence microscopy images of C2-arrested control and KS-induced (rapalog) schizonts of VPS16 parasites expressing APH-mCherry (cytosolic surface microneme protein). Nuclei stained with DAPI. Scale bars: 5 μm. (E) Immunofluorescence images of C2-arrested control and KS-induced (rapalog) VPS16 schizonts expressing EBA175-mCherry probed with anti-RAP1 (rhoptry luminal bulb protein) and anti-mCherry (RFP) (EBA175, microneme transmembrane protein). Nuclei stained with DAPI. DIC, differential interference contrast. Scale bars: 5 μm. Enlarged micrographs (zoom: 600x) of the indicated white boxes are shown right to visualise apical co-localization of both organelle markers (yellow arrows) and PV (light blue arrows) of RAP1 in contrast to the internal staining of EBA175 mCherry in KS-induced schizonts. (PDF) [file ppat.1013053.s005.pdf]
