# Supplementary material for: HOPS/CORVET tethering complexes are critical for endocytosis and protein trafficking to invasion related organelles in malaria parasites
Source: PLoS Pathog. 2025 Apr 8;21(4):e1013053. doi: 10.1371/journal.ppat.1013053 (PMC12011295; doi:10.1371/journal.ppat.1013053)
Supplement: S1 Table — Saccharomyces cerevisiae, Homo sapiens, Drosophila melanogaster and Tetrahymena thermophila. Accession numbers are indicated for Plasmodium falciparum and Toxoplasma gondii. NI: not identified. (PDF) [file ppat.1013053.s006.pdf]

S1 Table. Comparative bioinformatics analysis of genes coding components of CORVET and HOPS complexes in *Plasmodium falciparum and Toxoplasma gondii. Saccharomyces cerevisiae, Homo sapiens, Drosophila melanogaster and Tetrahymena thermophila.* Accession numbers are indicated for *Plasmodium falciparum* and *Toxoplasma gondii.* NI: not identified.

**HOPS/CORVET subunit**	***P. falciparum* Plasmo DB**	***T. gondii* homologue**	***S. cerevisae***	***Homo sapiens***	***T. thermo-phila***	***D. melano-gaster***
**VPS 3**	PF3D7_1423800	not identified	VPS3	TGF-BRAP1	VPS3	Absent
**VPS 8**	PF3D7_0916400	TGME49_289520	VPS8	VPS8	VPS8a	VPS8
**VPS11**	PF3D7_0502000	TGME49_230220	VPS11	VPS11	VPS11	Absent
**VPS16**	PF3D7_123900	TGME49_320670	VPS16	VPS16A VPS16B/ SPE-39	VPS16	VPS16
**VPS18**	PF3D7_1309700	TGME49_289730	VPS18	VPS18	VPS18	VPS18
**VPS33**	PF3D7_0935200	TGME4 9_295000	VPS33	VPS33A VPS33B	VPS33	VPS33
**VPS39**	PF3D7_1423800	TGME49_ 315530	VPS39	VPS39-2/TRAP1	Absent	Absent
**VPS41**	PF3D7_0916400	TGME49_ 224270	VPS 41	VPS 41	Absent	Absent
**VPS45**	PF3D7_0216400	TGME 49_271060	VPS45	VPS45	Absent	VPS45
**Beach -domain containing protein**	PF3D7_1124100	TGME49_263000	NI	NI	NI	NI
