# Supplementary material for: HOPS/CORVET tethering complexes are critical for endocytosis and protein trafficking to invasion related organelles in malaria parasites
Source: PLoS Pathog. 2025 Apr 8;21(4):e1013053. doi: 10.1371/journal.ppat.1013053 (PMC12011295; doi:10.1371/journal.ppat.1013053)
Supplement: S2 Table — (PDF) [file ppat.1013053.s007.pdf]

SUPPLEMENTARY TABLE S2
OLIGONUCLEOTIDES USED IN THIS STUDY

Primer Identification	Sequence 5-3	Cloning purpose
VPS3 fw NotI	CTCGgcggccgctaaAATGGACCAAAGAAATATAATAAAAAAAAAC	pSLI VPS3-2xFKBP-GFP-2xFKBP__nmd31xNLSFRBT2AhDHFR
VPS3 rv Avr II	TCCTcctaggCTCATCGTATTTCTCAATGCAATG	pSLI VPS3-2xFKBP-GFP-2xFKBP__nmd31xNLSFRBT2AhDHFR
VPS11 fw NotI	CTCGgcggccgctaaAATCAATCAAACAAACAATCAATCAATCAAAC	pSLI VPS11-2xFKBP-GFP-2xFKBP__nmd31xNLSFRBT2AhDHFR
VPS11 rv AvrII	TCCTcctaggTTTTGGTGTAATTCCATAGGAAAGG	pSLI VPS11-2xFKBP-GFP-2xFKBP__nmd31xNLSFRBT2AhDHFR
VPS16 fw NotI	CTCGgcggccgctaaGTTACTTAAATTGGCTAATTATAGATTAG	pSLI VPS16-2xFKBP-GFP-2xFKBP__nmd31xNLSFRBT2AhDHFR
VPS16 rv Avr II	TCCTcctaggTCTTATGTTTGATATGGCATCCG	pSLI VPS16-2xFKBP-GFP-2xFKBP__nmd31xNLSFRBT2AhDHFR
VPS18 fw NotI	CTCGgcggccgctaaCAACAAACTAATAGAAGAAAATATC	pSLI VPS18-2xFKBP-GFP-2xFKBP__nmd31xNLSFRBT2AhDHFR
VPS18 rv AvrII	TCCTcctaggGTCATTTGAAATATTCCATGTATC	pSLI VPS18-2xFKBP-GFP-2xFKBP__nmd31xNLSFRBT2AhDHFR
VPS33 fw NotI	CTCGgcggccgctaaTAATAATATAATAAACAAAACGATG	pSLI VPS33-2xFKBP-GFP-2xFKBP__nmd31xNLSFRBT2AhDHFR
VPS33 rv AvrII	TCCTcctaggCTCCATTGATTGTAAAAATTTC	pSLI VPS33-2xFKBP-GFP-2xFKBP__nmd31xNLSFRBT2AhDHFR
Vps3_fw_5prime	CCAAGAGTTATACAACGGG	Integration Check VPS3-2xFKBP-GFP-2xFKBP
Vps3_rv_3UTR	CAAAAACAAGAATGTAGTG	Integration Check VPS3-2xFKBP-GFP-2xFKBP
VPS11 intragen fw 5´	GTTCAAAAAAAAAAAAATTATATCAATGG	Integration Check VPS11-2xFKBP-GFP-2xFKBP
VPS11 rv 3´	CATTTTATATAAGGTTTAAATTAAATCATGTC	Integration Check VPS11-2xFKBP-GFP-2xFKBP
Vps16_fw_5prime	CAATTATGTAGTGCTATAACGG	Integration Check VPS16-2xFKBP-GFP-2xFKBP
Vps16_rv_3UTR	CCTACAAATATAAGGTATC	Integration Check VPS16-2xFKBP-GFP-2xFKBP
Vps18_fw_5prime	GAAATGGAAATATGCAATATG	Integration Check VPS18-2xFKBP-GFP-2xFKBP
Vps18_rv_3UTR	GTTTCTTTTCTGTGTTGTTACG	Integration Check VPS18-2xFKBP-GFP-2xFKBP
AP3-µ fw1 Gibson	GTTTTTTTTAATTTCTTACATATAACTCGAGATGCTGGATTTATTTTGTATTTATTCGTC	pcrt-AP-3 mScarlet
AP3-µ rv1 Gibson	GTGAATTATTACATTTTTATTATTTAGAG	pcrt-AP-3 mScarlet
AP3-µ fw2 Gibson	CTCTAAATAATAAAAATGTAATAATTCAC	pcrt-AP-3 mScarlet
AP3-µ rv2 Gibson	CATAATCTGGATTATCATATGGATAACTTGTCCTAGGTAGCCTAAATTCTATGTTATTATATATG	pcrt-AP-3 mScarlet
crt-GBP $^{1-108}$ fw	CATATAACTCGACCCCGGGATctcgagATGCGACTTTCTAAAGTATCTGA	pcrt-GBP1-108 mScarlet
crt-GBP $^{1-108}$ rv	CTGCCTCACCCTTACTCACCATcctaggTCTACTTAATATTGCTCCTGC	pcrt-GBP1-108 mScarlet
APH_KpnI_fwd	GCGCggtaccATGGGAAATACAGTTCCATATAATT	pama1-APH-mCherry
APH_AvrII_rev	GCGCcctaggCTTCATACTCATAATTTTTCTAAAGG	pama1-APH-mCherry
Rab5a_ KpnI fw	GGGggtaccGAAAAGAAAAGTAGTTATAAAACAGTTTTATTAGG	pcrt-mScarlet-Rab5a
Rab5a_ XmaI rv	GGGcccgggTTAACAACATCCTTTTTTTGAAAGTG	pcrt-mScarlet-Rab5a

Restriction sites are indicated in small caps. Overlapping regions for Gibson cloning are underlined
