# Supplementary material for: HOPS/CORVET tethering complexes are critical for endocytosis and protein trafficking to invasion related organelles in malaria parasites
Source: PLoS Pathog. 2025 Apr 8;21(4):e1013053. doi: 10.1371/journal.ppat.1013053 (PMC12011295; doi:10.1371/journal.ppat.1013053)
Supplement: Attachment — Submitted filename: Cover letter and response to reviewers.docx [file ppat.1013053.s009.docx]

Bernhard Nocht Institute for Tropical Medicine

P.O. Box 50 06 40, 22706 Hamburg, Germany

**Prof. Dr Tim Wolf Gilberger**

Head Cellular Parasitology Dept.

Bernhard-Nocht-Str. 74

20359 Hamburg | Germany

Tel	+49 40 8998 87600

Tel	+49 40 285380-270

Fax	+49 40 285380-265

gilberger@bnitm.de

Hamburg, 2025-01-29

Dear Editors,

Please find enclosed our revised manuscript entitled “**HOPS/CORVET tethering complexes are critical for endocytosis and protein trafficking to invasion related organelles in malaria parasites” PPATHOGENS-D-24-02339** for consideration as a publication in PLOS Pathogens.

We highly appreciate the comments and suggestions of the reviewers and are grateful for the constructive criticism. We also like to thank you for the extension of the re-submission date. We used the time to carefully edit the manuscript and the figures according to the reviewer’s suggestions. We also include the following additional data to address the reviewers suggestions:

1. Confocal microscopy of the VPS18-APH mCherry cell line to better visualize the presence of the micronemes upon KS of the VPS subunits despite defective cargo trafficking (Fig. 6 G).
2. Imaging and quantification of the efficiency of the knock sideways in the different cell lines (Fig. S2A-E)
3. A bloated vacuole assay and better imaging (magnifications) with the VPS18-GBPmScarlet line to confirm the content and endocytic nature of the vesicles that accumulate upon knock sideways (Fig. 3D,F, S4E).
4. A detailed discussion of the knock sideways system to support that the phenotypes observed are the result of the mislocalization of the subunit and not because of secondary unrelated events due to the accumulation of proteins in the nucleus.
5. Additional co-localization of HOPS/CORVET with Rab5a with a newly generated mScarlet-Rab5a expressing reporter parasite line using a different promoter for its expression (Fig. 3F).
6. Labelling and magnifications of typical and aberrant localization of rhoptries and microneme proteins after HOPS/CORVET knock sideways (Figure 5, 6 and S5).

We would be grateful if you would consider our revised paper for publication.

In the following, each point raised by the reviewer is presented in *italics*, followed by our response (blue) and the amendments introduced into the revised manuscript.

1. Reviewer #1: *Mesén-Ramírez* et al. *report studies of the HOPS/CORVET tethering complexes in the malaria parasite, P. falciparum. They use the knock-sideways DNA transfection technology to tag and study VPS11, VPS16, VPS18, and VPS3 components of these complexes; similar tagging of VPS33 was unsuccessful, suggesting that that component does not tolerate the tag. VPS3 knockdown produced a more modest growth defect than seen with the other components, so it was not further characterized. Knockdown of VPS11 and VPS18 compromised both intracellular parasite maturation (through compromised endocytosis of hemoglobin and fusion to the DV) and merozoite invasion (through compromised effector protein delivery to rhoptries and micronemes). The primary effects of VPS16 knockdown related to trafficking of proteins to rhoptry and microneme organelles and defective merozoite invasion. The studies are well-executed and involve a large number of relatively complicated DNA transfections and high-quality imaging studies.*

We are grateful for these appreciative comments and the thorough summary of our study.

Reviewer #2: *The study by Mesen-Ramirez and colleagues investigates the function of HOPS/CORVET tethering complexes in Plasmodium falciparum, The investigators use a combination of genetic and cell biologic techniques to demonstrate as essential role for this complex during the asexual stage of parasite replication. The SLI system shows a strong growth defect for three of the HOPS/CORVET core components (VPS11,16, and 18). The use of the bloated DV assay is quite clever and demonstrates a clear disruption of the endocytic pathway for VPS11. Additional colocalization studies with well-studied cellular markers further confirms the importance of the HOPS/CORVET complex for host cell cytoplasm endocytosis. Also, in very exciting data, the team demonstrates an important role for the HOPS/CORVET complex in trafficking of materials to the rhoptries and micronemes. Overall, this is a very neat paper without major weaknesses. Some suggestions, none of which are essential, are included below.*

We thank reviewer two for the detailed descriptions and appreciations of our findings.

2. Reviewer #3: *Mesén-Ramírez and colleagues investigate the functions of conserved elements of the HOPS/CORVET complexes in vesicle trafficking events of blood-stage Plasmodium. They use a knocksideways strategy to perturb protein function and observe strong phenotypes where either early growth is disrupted and linked to incomplete delivery of endocytosed host cytosol to the digestive vacuole, or failure to generate infective merozoites where key rhoptry and microneme proteins are seen to lack typical location patterns. Overall, the work identifies elements of HOPS/CORVET core proteins in both of these processes, and discerns a difference for VPS16 that might be less involved in endocytosis. The approaches taken to verify the phenotypic observations are extensive and generally well executed. The absence of TEM-based ultrastructural analysis means that some of the conclusions of cellular defects are more indirect, but most are warranted with some caution. My main query is the interpretation of the use of mislocalistation of these proteins as equal to ‘inactivation’ or neutral loss of function, and I think that further discussion of this technology and its possible caveats should be clearly presented to the reader.*

We also would like to thank reviewer 3 for the constructive criticism and critical appreciation of the work. Below, we provide a short response to the reviewer’s technical questions, with more detailed information provided in the point-to-point discussion section as indicated:

First, we agree that the knock sideways strategy to study function of the HOPS subunits by recruiting the subunit to a different cellular compartment (in our study, the nucleus) does not necessarily mean a complete inactivation or neutral loss of the protein function and might have unpredicted secondary effects. We use the term inactivation for simplification purposes but the actual event of knock sideways is indeed the mislocalization (anchor away) of the protein from the physiological site of action. We discuss this in more detail in point 8 but would like to note here that this system has proved to be well-suited for the rapid inactivation of vesicle trafficking factors not only in *Plasmodium* but also in model organisms (Hirst et al., 2012; Robinson and Hirst, 2013)

Second, we certainly acknowledge that TEM (in particular a cryoEM) derived data set could contribute to a more precise visualization of the phenotypes (discussed in point 4 and 9). But we also would like to note that we have used different fluorescent imaging techniques (e.g. U-ExM, confocal) with a broad range of marker cell lines and quantitative analysis approaches to maximise experimental support for our interpretation of the phenotypes.

**Part II – Major Issues: Key Experiments Required for Acceptance**

Reviewer #1:

3. *The findings are in line with similar studies in other eukaryotes. Notably, they parallel studies in Toxoplasma gondii, though some modest differences are described in the Introduction. As such, the Discussion would benefit significantly from a more clear description of what findings are unique to P. falciparum and related malaria parasites. This could include insights gained about evolution of subunit sequence and structural similarities/differences in the tethering complexes.*

We appreciate this suggestion and have include now a more detailed paragraph in the discussion (Line 403-410; 437-450; 509-534)- to discuss differences in the composition and function of the complexes that might be unique to malaria parasites and some insights from other organisms. The composition of the complexes in the parasite besides the core components remains unclear and can only be revealed using proximity proteomic analysis (not performed in this study) to identify the corresponding accessory factors that associate to the core at different time points (e.g. trophozoites (endocytosis) vs schizont (organelle trafficking)).

4. *Although the immunofluorescence and expansion microscopy images are of high quality and generally support the authors’ interpretations throughout, I wonder whether the manuscript would benefit from TEM imaging of trophozoites to show accumulation of vesicles in the cytosol that fail to fuse with the nascent DV (e.g. with and without E64 treatment) and of schizonts to establish effects on secretion to invasion-associated organelles.*

We appreciate the observation of the reviewer and we agree that TEM is a powerful method that might benefit the visualization of endosomal structures that failed to fuse to the FV. Due to resource and time limitations we, in response to this comment (and also the comment from reviewer 3), proceeded with additional analyses using ultrastructure-expansion microscopy (U-ExM), a well-established method in *Plasmodium falciparum*. U-ExM allows for a quick, flexible, quantifiable and inexpensive method for observation of control and mutant parasites with resolution near comparable to EM. We also would like to point out that we analyzed the nature of these vesicular structures by alternative and complementary methods such as i) the bloated vacuole assay (**3A**), ii) the assay with the exported mCherry reporter (**Fig 3D and E**) and iii) the PI3P marker P40XP to label primary endosomes and the DV membrane (**3C**). Using these complementary approaches, we could confirm that these vesicles contain host cell cytosol and are primary endosomes that cannot fuse to the digestive vacuole membrane and hence do not deliver hemoglobin to the DV.

To follow the suggestion of the reviewer we performed the experiment using the line VPS18- GBPmCherry in the presence/absence of E64 and imaged these conditions (now **Figure 3E**). We clearly can observe the presence of a bloated vacuole and no vesicles in the controls, while the knock sideway parasites do not swell the DV and accumulate vesicles containing the mCherry reporter. As discussed below in point 5 we also lysed the infected red blood cells with saponin to release the exported mCherry protein from the host cell cytosol and visualize better the host cell containing vesicles in the parasite cytosol (**Figure S4E**).

To establish the effects on trafficking to invasion-associated organelles, we took advantage of several organelle markers to assess proper trafficking and U-ExM to visualize organelle morphology and measure the changes in volume caused by any cargo trafficking defect (**Figure 7**). We now provide confocal microscopy of the micronemes (**Figure 6G**) and magnifications of the different phenotypes to support our statements (**Figure S5**). Also, we labelled typical and aberrant localizations to help the reader to distinguish the phenotypes. See point 6 and 9

Reviewer #2:

These are not really "major" but included here nonetheless because they rise above typographical errors.

5. *For 3B, the scale of the graphs makes the phenotype difficult to appreciate. I suggest that the scale be changed to 10 and the parasites with >10 could be indicated by “pinning” to the top of the graph. For 3D, the colocalization is also difficult appreciate. I recommend including a zoomed region on in this figure to allow the better appreciation of the colocalization. If this colocalization could be quantitified, that would be an excellent addition as well.*

We thank the reviewer for these suggestions and we modified the figure accordingly by changing the scale to 15 vesicles for VPS11 and 18 and to 10 vesicles for VPS16.

We include in **Figure 3D** a zoom of the vesicles containing mCherry signal as an indication for the content and origin of these vesicles. Due to the small size of these structures and the lower mCherry signal in these cytosolic structures in the parasite cytosol compared to the cytosolic bulk in the host cell, we could not reliably quantify the localization in comparison to controls, where these vesicles are lacking or in low numbers. We now provide images of these parasites when we lysed the red blood cell membrane using saponin (a detergent that lyse selectively the RBC membrane but not the parasite membrane and leads to release of the host cell cytosol (containing the mCherry reporter) (**Figure S4E**). This helps to visualize that the vesicles in the parasite cytosol indeed contain host cell cytosol.

6. *For figure 5 and 6, it would be helpful to show and label examples of normal/apical and aberrant localization. For RAP1, the mislocalization is more obvious. For VPS11/RON12, it is more subtle and would benefit from some additional examples with labeling. This is an important finding and is quite subtle. I realize that this is shown better in the supplemental figure. Thus, a simple solution would be to include some of the supplemental data in the main figure itself. The data in figure 7 are very convincing. It might make the story stronger to include the respective panels in figure 5 and 6, instead of having a separate figure 7.*

To address this point, we now label (using arrows) a typical apical localization (yellow) and an aberrant (blue) for the different subunits in **Figure 5, 6 and S5A, B &E**). We observed for the markers analysed in VPS11-KS mostly a mixed phenotype (as quantified from the number of cells) of apical and diffuse localization suggesting that the knock sideways of this subunit had less impact in protein trafficking to the organelle or the efficiency of the knock sideways for this subunit in schizont was lower than for the other subunits. We now include in **Figure S5B** images with magnifications of VPS11-RON12 where the mis-trafficking of the rhoptry protein is more evident.

We also added a new panel in **Figure S5E** to show the mis-trafficking of RAP1 and EBA175 in the same schizonts. RAP1 localized to the parasite periphery (likely distributed in the PV) and EBA within the merozoites in contrast to the punctate apical localization in control.

Because of space restrictions in Figure 5, in order to show more examples we include the magnifications in the supplementary Figure 5 (**S5A,B**).

We agreed with the reviewer that the data in Figure 7 complemented the results show in Figure 5. However, because of the dimensions of the data and micrographs the suggested merge of these data sets was not really satisfying. We hope that that as it stands this is acceptable for the reviewer.

7. *Many of the microscopy images, especially the live cell images, look like maximum projections or even widefield images of the entire parasite. I wonder if the phenotypes would be more apparent if single z-slices were shown or a subset of z-slices. This is a style comment, so it is up to the investigators*

The microscopy images correspond to differential interference contrast images and we agree that we did not show stacks for these images. We had to mitigate the low expression of the VPS subunits across the cycle that challenged microscopy analysis using confocal microscopy because of photobleaching.

Nevertheless, to address this comment we have now tried quite extensively and were able to obtain satisfactory confocal microscopy images to visualize the morphology of organelles using VPS11-AMA1 (**Figure S5C**) and VPS18-APH (**Figure 6G**).

We also improve the expression of Rab5a in **Figure 3F** using a plasmid with a different promoter (crt) and now we can better visualize the co-localization of this marker with HOPS/CORVET proximal to the vesicular foci but not at the DV. We changed it accordingly in the main text (Line 252-253)

Reviewer #3:

8. *The study uses a mislocaliser with a nuclear localisation signal. I assume that this means that the mislocaliser is constantly cycling between the cytosol and nucleoplasm so that it is available to bind to its partner when the rapalog is added. So, what happens next? Is the protein of interest fully delivered to the nucleoplasm, or does it get stuck at the nucleopore complex, or does it cycle back and forth between the nucleus and cytosol as the mislocaliser is presumed to. Does it dissociate from the mislocaliser, and if so is the frequency of this different for different proteins? Would the kinetics of different cargo proteins (POIs) be different according to how they might dissociate in the nucleus? Where the site of function is known, mislocalising to the nucleus/nuclear pore complex/nuclear envelope might be sufficient to disrupt function. But if vesicle-tethering machinery is being delivered to another part of the endomembrane system (nuclear envelope here) and potentially in proximity to other compartments then could new and aberrant tethering events be promoted and more general disruption of the endomebrane ensue? No data is shown for the actual location of the bulk of the mislocalised POIs, and even so it would be hard to determine the location of minor populations. All of these uncertainties make interpretation of the mutants more complex than ‘inactivations’ as might be made for protein knockdown or knockout. Comparing the effects between proteins is potentially equally problematic here if different extents of effective ‘inactivation’ might occur. These issues need to be discussed openly and considered in the weighing up of support for the conclusions reached. I think that the approaches taken are sophisticated and address the lack of other fast mutant generators in Plasmodium, but they are not without their limitations and complexities also, perhaps especially so were the proteins of interest might be involved in many interactions within the cell. Some of the conclusions need to be treated as new untested hypotheses that warrant further investigation now.*

The mislocalizer (a nuclear localization signal appended to an FRB domain) once synthesized (under the nmd3 promoter) is trafficked to the nucleoplasm. To demonstrate the effectiveness of this system we now include data in S2A for the VPS11-2xFKBP-GFP-2xFKBP line expressing an NLS-FRB-mCherry that allows for a better visualization of the mislocalizer in the nucleus (in the original KS lines used in this study the mislocalizer 1xNLS-FRB has no tag). When rapalog is added, the dimerization of the FRB-FKBP domains leads to the recruitment and transport of the protein of interest to the nucleus where it remains irreversibly associated to the NLS-FRB molecules away from the site of action. Based on the localization of the VPS11-2xFKBP-GFP-2xFKBP line expressing an NLS-FRB-mCherry and previous reports (*e.g.* Birnbaum et al., 2017, Jonscher et al., 2019, Birnbaum et al., 2020, Geiger et al., 2020, Kimmel et al, 2023, Sabitzki et al., 2024) it is expected that the protein of interest is totally or partially transported to the nucleoplasm (not stuck to the nuclear pore at the nucleus membrane) and does not cycle between the cytosol and nucleus as long as rapalog is present. Only upon removal of rapalog would newly synthesized protein localize to the site of action. Initial validation of the system showed for two proteins (Rab5a and Kelch13) that the general phenotype of the NLS knock sideways was similar to diCre based conditional gene knock outs (Birnbaum et al., 2017). Many targets have since been mislocalised to the nucleus, and so far, there has been no indication for an unrelated negative effect due the presence of the target in the nucleus or residual function due to tethering to the nuclear periphery of the parasite (Birnbaum et al., 2017, Jonscher et al., 2019, Birnbaum et al., 2020, Kimmel et al, 2023, Sabitzki et al., 2024) (now explained in Line 135-138). We do not claim here that the system has no drawbacks, but we are confident that the findings in this paper are not a result of indirect effects of the targets in the nucleus or residual activity in the nucleus periphery.

The dissociation constant of the protein does not depend on the protein itself but on the number of FKBP domains fused to it (Birnbaum et al., 2017). The expression profile of the protein and the promoter timing might influence the amount of protein trafficked to the nucleus or localized to the site of action. Previous studies have demonstrated the efficiency of the 1xNLSFRB mislocalizer for endolysosomal factors (Birnbaum et al., 2017; Jonscher et al., 2019; Geiger et al., 2020, Sabitzki et al., 2024), which allows for a rapid recruitment after 1h incubation. It can be assumed that the interaction FKBP-FRB is stable and irreversible as long as rapalog is present in culture. Attempts to reverse the interaction have failed, as wash out of rapalog did not lead to a release of the target protein from the nucleus in a time frame of hours and likely only newly synthesised protein will localize to the natural compartment and can restore function (unpublished observations from Spielmann lab (BNITM, Hamburg) and ours). We therefore conclude that the target protein amounts sequestered to the nucleus in this study are unlikely to contribute to its normal physiological function.

In the case of the HOPS/CORVET subunits, the mislocalization to the nucleus is expected to inactivate the function of the protein/complex by removing it from the site of action (cytosol, food vacuole membrane, vesicular structures) (Line 130-132). We agree with the reviewer that this is not exactly an inactivation or knockout/knock down of the protein and is instead a mislocalization to another compartment where the protein is not functional. However, this approach is factually similar to an inactivation, as the protein is not present at the natural compartment and therefore not available to perform its function. For the sake of readability, we simplify this with the term ´inactivation` and define this on first use (Line 142) as has been done in previous studies.

We cannot exclude that the transport of the tethering subunits to the nucleus might lead to secondary tethering events in the nucleus. However, this is unlikely in the nucleus since the tethering and membrane fusion is a coordinated event that require specific Rab and SNARE proteins absent in the nucleoplasm.

We now included micrographs showing the mislocalized VPS subunits upon addition of rapalog and quantification for those where it was partial (Fig S2A-E). The presence of minor pools of protein not mislocalized/inactivated depends on the efficiency of the mislocalization. As HOPS/CORVET are protein complexes, inactivation of individual subunits might still leave some residual active subunits that still can perform their functions, however the striking growth defect observed for the core subunits indicates that this pool still present outside the nucleus is likely not functional. Using the modified SLI method (Sabitzki et al., 2024) we ensure that all parasites harbour the nuclear mislocalizer (the plasmid is integrated in the genome and not expressed episomally from a second plasmid)(Line 132-135). We cannot exclude differences in the mislocalization in the different parasite stages and individual parasite differences in term of mislocalizer expression. However, the strong growth defect (> 97%) for the three core subunits and the evident phenotypes suggest that the knock sideways is highly efficient in these parasites and leads to the expected inactivation. We now provide images and quantifications of the mislocalization (Figure S2B-E) that support that the system is efficient for our purposes (Line 151-159). There is no clear difference in the mislocalization efficacy in the different cell lines and hence this allows for a comparison of the different phenotypes (now Line 157-159)

Results using a plasma membrane mislocalizer (Lyn -mCherry; Birnbaum et al., 2017) in VPS11 resulted in a severe growth phenotype comparable to the nuclear mislocalizer, supporting that the phenotypes analysed are not caused by the nuclear relocation of the factors to the nucleus but specifically by the knock sideways of VPS11. This line was not further analysed in this study, only shown here to confirm that the inactivation via KS of VPS11 is independent of the compartment where the subunit is mislocalized.

VPS11-2xFKBP-GFP-2xFKBP + Lyn FRB mCherry

It should be noted that a knockdown using the glms system was tested for phenotype with the subunits, however, this yielded only a minor growth defect due to incomplete protein knockdown upon addition of glucosamine. The big advantage of the knock sideways in this study is its versatility for stage-specific rapid inactivation of the HOPS/CORVET subunits at different time points. This allowed us to observe the phenotypes in a much more targeted fashion, yielding the interesting stage-specific phenotypes. In contrast, a knock-down or knock-out system requires a broader time frame for near-complete protein depletion and limited the temporal phenotypic characterization.

9. *It is a shame that TEM was not used and that the presence and appearance of membrane-bound organelles was assessed by indirect methods of protein association. If resources allowed, I’d recommend providing direct data on the presence of micronemes and host cytosol-filled vesicles.*

We agree that TEM data (in particular cryoEM derived data) could be of some benefit to the assessment and visualization of the membrane structures/vesicles accumulating as a result of the knock-side ways. Due to resource and time limitations, we were unable to undertake the required sample preparation, imaging and analysis in the time frame for this review. As an alternative, we proceeded to analyse these mutants with U-ExM (near TEM level resolution) in conjunction with other assays as described in point 4.

In contrast to the analyses in *Toxoplasma gondii* or other *Plasmodium* stages, visualization of micronemes in *Plasmodium* merozoites appears also to be challenging using U-ExM and TEM as it cannot distinguish structures certain vesicular structures such as dense granules, exonemes and micronemes in the absence of specific organelle marker antibodies. The lack of suitable antibodies in our lab restricted the possibility of analysing micronemes and hence we proceeded to use APH mCherry (bound peripherally to the microneme membrane) as a marker for the integrity of the micronemes after KS of the VPS subunits. We now include data from VPS18-APH mCherry (**Figure 6G**) to support the phenotype observed (defect on transport of secretory proteins but intact micronemes) using live confocal microscopy to show the presence of micronemes with no apparent alteration.

**Part III – Minor Issues: Editorial and Data Presentation Modifications**
Please use this section for editorial suggestions as well as relatively minor modifications of existing data that would enhance clarity.

Reviewer #1: (No Response)

Reviewer #2: (No Response)

Reviewer #3: Minor Comments that would aid the clarity of the report and address some over-interpretations of the data presented during the narrative.

10. *Line 107-110: In so far as the yeast/mammalian complexes are considered canonical, then defining presence and absence in apicomplexans might be useful. But this comparison doesn't allow any assessment of what might be typical of eukaryotes, including the potential ancestral state. Only two lineages are being compared here: apicomplexans against opisthokonts, so the limitations of this comparison should be acknowledged.*

We appreciated this observation and agree that our initial bioinformatic analysis was too focused. Due to the extreme variability of the composition of the complexes in eukaryotes and the lack of conservation between the subunits of the different organisms we focused our BLAST analysis on the related apicomplexan *T. gondii*. We now include more data on the composition of HOPS/CORVET in yeast, mammalian cells and metazoans in Table 1.

11. Line 125-6: The logic of why the function of the core HOPS/COVET subunits would be 'inactivated' by mislocalisation needs to be spelled out, including any assumptions that are being made here. Is it clear that these proteins enter the nucleoplasm where they presumably can't function. Could some be mislocalised to nuclear pores but induce some secondary changes in HOPS/COVET activity? Would this KS proteins be expected to relocate other elements of these proteins with them? While phenotypes might be observed with this KS approach, the possible mechanics of this needs to be considered beyond 'inactivation'.

We agree and acknowledge that secondary effects might result from the relocation of HOPS/CORE subunits to the nucleoplasm (see discussion above, point 8). We now include a sentence to acknowledge this limitation (Line 135-138). To exclude this the confirmatory approach will be complementing these mutants with the corresponding VPS subunit but the gene length (>4000 bp) limited a complementation strategy in this study. Previous studies on endolysosomal factors relocated to the nucleus such VPS45, Rab5a and Rabenosyn 5 (Birnbaum et al., 2017; Jonscher et al., 2019; Sabitzki et al., 2024;) support (through complementation) that the phenotypes observed using the knock sideways strategy are specific for the removal of the protein from the site of action and not because of the localization to the nucleoplasm. See point 8

12. *Line 138: '>97%' needs confidence values given the precision of the value stated.*

The growth defect for every subunit is now stated with confidence values. (Now Line 143-144)

13. *Line 141: is it known if the KS approach is equally efficient for all proteins. If not, then comparing phenotypes between proteins might be difficult to do. Again, the question of how tightly KS can 'inactivate' is relevant.*

We acknowledge this reviewer´s concern and provide arguments to support the usefulness of the KS approach. We now provide in Figure S2B-E quantifications of the efficacy of the knock sideways in the different cell lines. Although some individual parasites display a partial localization (nucleus and vesicular foci), the subunit of interest was mislocalized to the nucleus in the overwhelming majority of the parasites counted (VPS3: 75.4±4.8%; VPS11: 59.2±31.5%; VPS16: 57.5±5.1%; VPS18: 68.8±6.4%) and the efficacy was similar for all subunits (See Fig. S2B-E). We add an explanatory sentence in line 151-159.

14. *Line 154: would you expect membrane vesicles to be persevered post Giemsa staining? While I agree there are white structures evident, I wonder if these can be concluded as vesicles. The ring-stage has a big whole in the middle by Giemsa, but as far as I understand this is never seen in TEM at this stage so is likely an artifact.*

The presence of white regions suggestive of vesicles in Giemsa smears of rapalog-induced parasites was only a preliminary indication that led us to further analysis using other microscopy and cell biology methods. Please see also point 4. We acknowledge that these Giemsa images are not conclusive of vesicles but these were quantified and confirmed using differential interference and fluorescence microscopy (BODIPY and DHE) (**Figure 3B and S4B**) and specific markers (P40XP) as well as the assay with the exported protein GBP that support the endocytic nature of the structures. At this point of the text we call them now cytosolic vesicle-like structures (Line 216, 225). In addition, the endocytosis phenotype observed in our mutants is comparable to other well-established phenotypes where these structures have been validated as vesicles (line 217-218) and were initially also evident as white areas in Giemsa smears (Jonscher et al., 2019; Sabitzki et al., 2024; Schmidt et al., 2023).

14. *Line 183: I guess TEM would be required to identify these as vesicles. As seen here, they are some form of occlusion in the cytosol, but 'vesicle' is just one possible interpretation. I think the authors need to be more objective in their description of their observations and be clearer about what are then untested hypotheses.*

EM based analyses with technique that allows the robust visualization of membranes would definitely help to better visualize these structures (please refer to point 4 and 9). As mentioned before, we used the BODIPY staining and the P40XP labelling to confirm that these structures are membrane-bound and correspond likely to vesicles (**S4B, D**). We called them first in line 216 & 225 vesicle-like structures to be careful with our initial statements. We could not also guarantee the preservation of these structures using TEM. Cryo EM would be optimal for these analyses but due to resource limitations we were not able to provide these analyses. Therefore, we have now tried to tone down our interpretation to avoid potentially misleading statements.

15. *Line 208: while vesicles might start to accumulate, their contents is speculative here and they could derive from some other process going awry here. TEM would be needed to show that they contain host cytosol.*

We agree with the reviewer that vesicles might result from other processes (e.g defect in membrane recycling, fragmentation of organelles), we confirm the nature of these vesicles as primary endosomes by labelling them with P40xP (indicative of PI3P on their cytoplasmic face) and confirming their content by expressing a mCherry exported protein localized to the RBC cytosol that-once reinternalized by endocytosis- will be contained in the endocytic vesicles as shown in Figure 3D, 3E and S4E. Please also refer to point 4.

16. *Line 212-3: could some general perturbation of endomembrane trafficking disrupt this PI3P assignment? TEM to look at vesicle contents is still likely better.*

We agree that fragmentation of the DV for example might also led to structures labelled with PI3P. We have now added a sentence (now line 235-236) to acknowledge this possibility. However, the results of the assay with the exported protein GBP-mScarlet showed that these vesicles containing host cell content are likely coming from the endocytic pathway rather than from a fragmented digestive vacuole. Similarly, the bloating phenotype speaks against a fragmentation of the DV, as only at the beginning of the assay hemoglobin starts to accumulate (through addition of E64), it is likely that the hemoglobin filled vesicles derive from newly formed endosomes, not a fragmentation of the DV (which typically does not contain undigested hemoglobin at the beginning of the assay). The reduction of the hemozoin size (Figure 3E and S4C) (not its fragmentation) as observed and the lack of DV swelling are rather indicative of a trafficking defect (Now line 241-243).

17. *Line 216: how is 'mostly adjacent to the DV' assessed? The DV is big, the cell is small . . .*

We agree that we did not provide any data to quantify the distance of the vesicles to the DV to assess proximity. It was based on a subjective observation that most of the vesicles were close to the DV. When we counted the number of vesicles localized next to the DV (hemozoin accumulation) or separated away from it, we found that 63,6% (VPS11) and 71,4% (VPS18) of the vesicles were found proximal/adjacent to the DV. We include these values now in the main text (line 230-231). Here the quantification:

18. *Line 267: why was it necessary to C2 arrest the cells and then release them from this. Could natural egress not be distinguished with the build-up or not of schizonts. Could the C2 treatment cause a secondary effect? Some justification for the experimental approach is warranted at least.*

As we mentioned in line 171-173 we used the reversible egress inhibitor C2 to prevent the rupture of schizont stage parasites and ensure that parasites were of the same age at the time of analysing invasion because of the apparent growth delay observed for VPS11 and VPS18. For the egress/invasion experiment we expected to start the experiments with age-matched control and KS-induced schizont parasites that upon removal of the compound, will initiate simultaneously the process of egress and invasion of new RBCs. This approach has been well established and used in several egress and invasion studies (Sherling et al., 2019; Alder et al., 2022; Liffner et al., 2022). It is demonstrated that C2 has no secondary detrimental effects when parasites are kept on the compound for time windows of 4-8 hours, due to the specific inhibition of the cGMP-dependent protein kinase G necessary for the activation of the egress cascade (Taylor et al., 2010).

19. *Line 290: it is not very clear what this interpretation of 'closer to the PV' means seeing that the apex of the parasites is already close to the PV. How is this assessment made? What are the 'internal structures' alluded to? This description is ambiguous.*

We agree that this description is ambiguous. RON12m Cherry appears to be distributed diffusely in the parasite periphery, around and in the nascent merozoites (probably the PV, consistent with the data of RAP1). The internal structures in the nascent merozoites we alluded to might be the vesicular structures that we observed in Figure 7 in expanded samples. We modified the sentence in line 309-310 and 315 and include more images and magnifications for RON12 in Figure S5A to allow the reader to better assess the phenotype. We also labelled typical and aberrant localization of the protein to visualize better the phenotypes

As discussed in point 6 we also added a new panel in **S5E** to show the mistrafficking of RAP1 and EBA175 in the same schizonts. RAP1 localized to the parasite periphery (likely distributed in the PV) and EBA within the merozoites in contrast to the punctate apical localization in control.

20. *Line 291: a changed pattern of RON12 doesn't necessarily imply that it is not reaching its destination, after all, the destination might have changed location. This is over-interpretation of the data at this point.*

We agree that at this point of the text this sentence is an over-statement and we have changed it accordingly in the main text (now line 311). RON12 is mis-trafficked and shows a localization that differs from the apical localization observed in controls (**5A-C and S5A**), although the rhoptries appear to be formed as confirmed in Figure 7.

21. *Line 319: is being in the cytosol and being close to the nucleus different?*

Close to the nucleus indicates an ER localization and cytosol indicates rather a diffuse pattern in the merozoites or the plasma membrane. We modified accordingly in the main text (Line 339-340) and showed now zooms of EBA175 for all KS lines in Figure S5B as well as a new panel in S5E to show the mistrafficiking of RAP1 and EBA175 in the same schizonts. The cytosolic localization probably corresponds to the vesicles that we observe in expanded samples.

22. *It is hard to rationalise how micronemes would still form as organelles if vesicular trafficking to them is blocked. And why SP-containing proteins would then appear to accumulate in the cytosol.*

We agree that the presence of micronemes after KS of HOPS-CORVET is intriguing. Based on the localization of APH-mCherry the organelles appear to form despite the disruption of the vesicular trafficking. We now provide further evidence using confocal microscopy of the VPS18-APH mCherry line (**Fig. 6G**). One explanation is that the organelles are already formed at the time point of induction (32-36 h.p.i) or that different tethering factors are involved in the biogenesis of the organelles and in the transport of protein cargo to them. In addition, the formation and function of vesicular organelles such as exonemes (based on the egress analysis) is not impaired upon inactivation of HOPS/CORVET, broadly suggesting that vesicular organelles still can form independent of HOPS/CORVET.

We acknowledge that it is unlikely that a SP-contaning protein accumulate soluble in the cytosol. From our results, SP-containing rhoptry luminal proteins (RON12, RAP1) appear to be distributed in the PV, around the nascent merozoites (probably following the classical default secretory pathway) or in internal structures (probably vesicular structures accumulating in the cytosol that cannot fuse to the organelles) (Line 309-310, 315, 468-469). SP-containing microneme transmembrane proteins (AMA1, EBA175) appear to remain trapped in the ER close to the nucleus or diffusely in the merozoite or at the plasma membrane (Line 339-340; 345-346; 490-491. See new Figure **S5A, B & E.**

We modified the text accordingly to avoid confusion.

Yours sincerely,

Prof. Tim Wolf Gilberger
